# Sagittal realignment osteotomy for increased posterior tibial slope after opening-wedge high tibial osteotomy: a case report

**DOI:** 10.1186/1758-2555-1-26

**Published:** 2009-11-26

**Authors:** Yuka Kimura, Yasuyuki Ishibashi, Eiichi Tsuda, Akira Fukuda, Harehiko Tsukada

**Affiliations:** 1 Department of Orthopaedic Surgery, Hirosaki University Graduate School of Medicine, Hirosaki, Japan

## Abstract

A 40 year old welder who underwent opening-wedge high tibial osteotomy for correction of alignment in a varus knee developed persistent pain with loss of knee extension. The posterior tibial slope increased from 9 degrees to 20 degrees after the osteotomy and caused the anteromedial knee pain and limited extension. The patient then underwent a revision osteotomy using a closing wedge technique to correct tibial slope. The osteotomy was performed, first from the medial cortex in the lateral direction, and second in the anteroposterior direction to remove the tibial bone in wedge shape and obtain full extension of the knee. The posterior tibial slope decreased to 8 degrees after the revision osteotomy and the patients returned to pain-free daily life. We reviewed this unique technique for correction of sagittal malalignment using a closing-wedge osteotomy for revision after opening-wedge osteotomy.

## Introduction

High tibial osteotomy (HTO) is a widely accepted surgical option to treat medial compartment osteoarthritis or varus malalignment of the knee. Strict criteria for patient selection, which are younger than sixty, knee flexion greater than 120 degrees, minimum lateral thrust and varus deformity less than 10 degrees, are essential for a good outcome [1,2]. Opening-wedge HTO has recently been popular because of its relatively safer procedures in which no dissection of the fibula or proximal tibiofibular joint is required, and therefore the peroneal nerve and lateral collateral ligament are left intact. The results of opening-wedge HTO are technically demanding and the successful outcomes in a long-term follow-up have been demonstrated in patients with appropriate angular correction [3,4]. Since the HTO is a realignment procedure in which the weight bearing axis is shifted from the pathological compartment to the other healthy compartment, much attention has been paid how to achieve accurate correction in the coronal plane [5]. Overcorrection and undercorrection of the coronal malalignment have been reported as the significant factors causing poor results. On the other hand, unintended change in the sagittal alignment has been produced in association with the correction procedure of coronal alignment in HTO. It was reported that closing-wedge HTO reduced the posterior tibial slope [6-8], whereas opening-wedge HTO increased it [5,8,9]. The change in the posterior tibial slope alters biomechanics in the tibiofemoral joint, especially in cruciate deficiency knees [10]. In cruciate intact knees, a small increase in the posterior tibial slope after opening-wedge HTO may have only subclinical effect and is asymptomatic in most of the patients. Sterett [11] reported that there was no apparent adverse effect on postoperative knee extension or functional knee score in spite of the significantly increase of posterior tibial slope by an average of 4 degrees after opening-wedge HTO. However, excessive increase could cause loss of extension and lead to tibiofemoral joint pain, and thus deteriorates postoperative clinical results.

We present a patient who underwent the closing-wedge osteotomy with unique techniques for treatment of flexion contracture and anteromedial knee pain that resulted from the increased posterior tibial slope associated with failed opening-wedge HTO. The patient was informed that the data concerning this case would be submitted for a publication, and gave his consent.

## Case report

A forty-year-old male welder with no past history of knee injury presented to another institution with left knee pain in September 2000. He underwent an arthroscopic partial menisectomy of the medial meniscus. In December 2004, 4 years after the primary surgery, he had a second arthroscopic partial menisectomy of the medial meniscus. However his left knee pain was not reduced. The radiographs of his left knee revealed a narrow medial joint space, femorotibial angle of 178 degrees, and posterior tibial slope [12] of 9 degrees. In January 2005, when he was 44 years old, he received an opening-wedge HTO using a Puddu plate with a 15-mm opening block and osteochondral transplantation of the medial femoral condyle. The femorotibial angle was corrected to 170 degrees after the osteotomy, while the posterior tibial slope increased to 20 degrees. The Puddu plate was removed at 18 months after the osteotomy; however, he still had severe knee pain.

**Figure 1:**
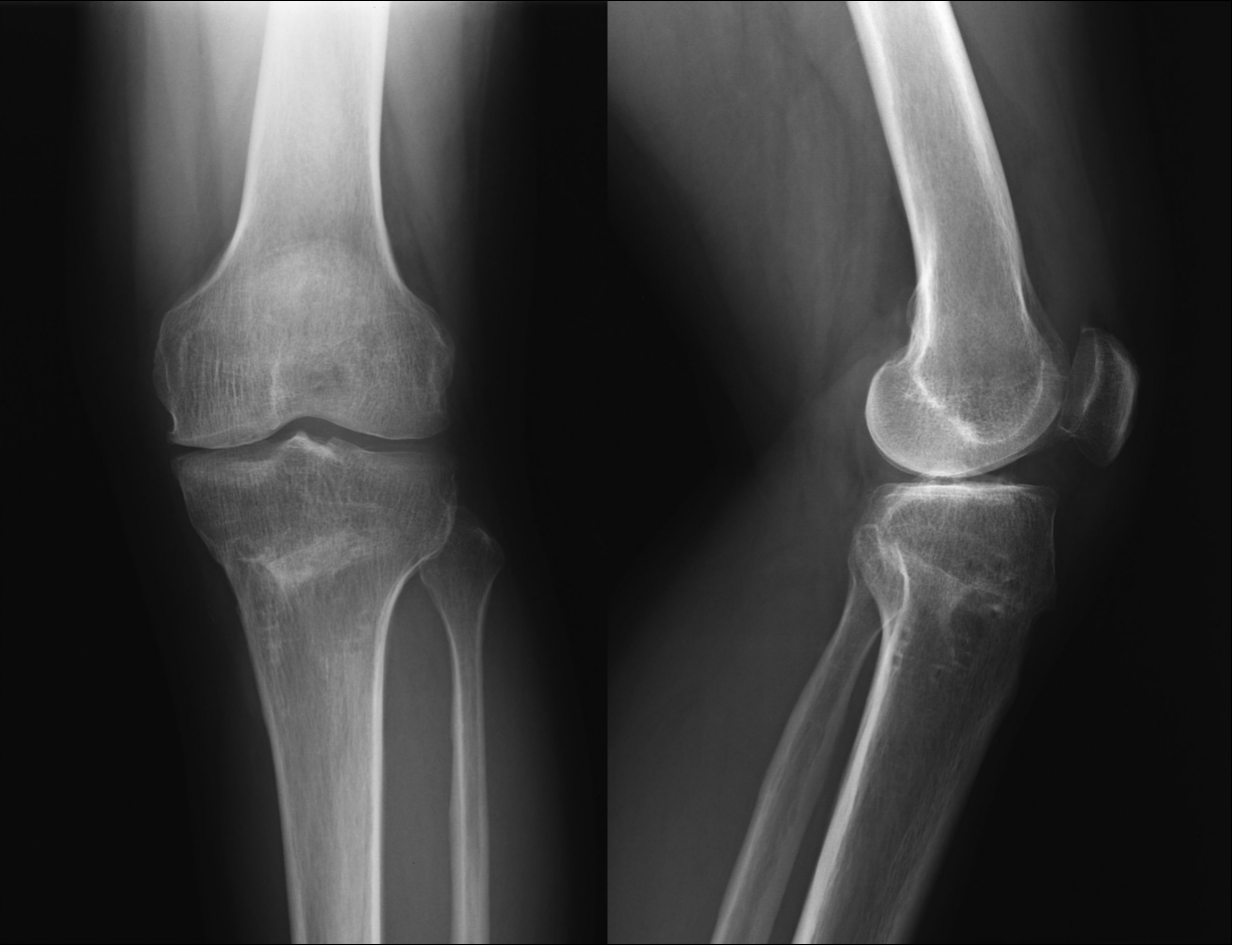
**Anteroposterior (left) and lateral radiographs (right) at 5 months after the primary osteotomy showing an increasing posterior tibial slope**. The femorotibial angle was 169 degrees and the posterior tibial slope was 20 degrees.

**Figure 2:**
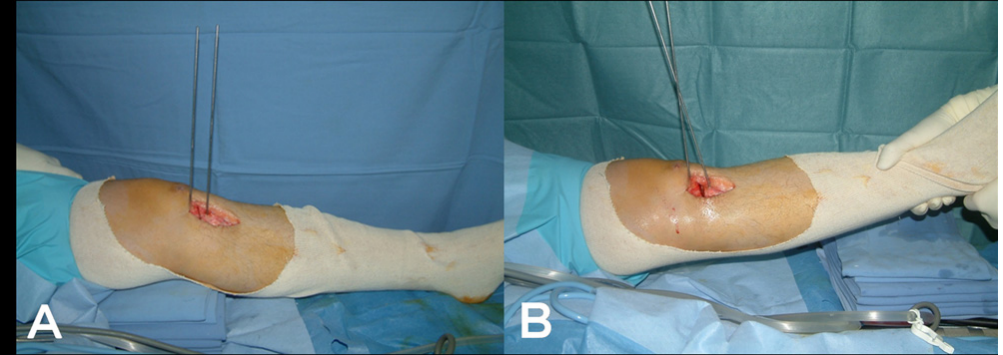
**Photographs showing the closing-wedge osteotomy**. The two parallel guide wires were inserted and used to monitor the sagittal alignment correction (A). At the end of the corrective procedure, full knee extension was obtained with 15 degrees of angular correction (B).

**Figure 3:**
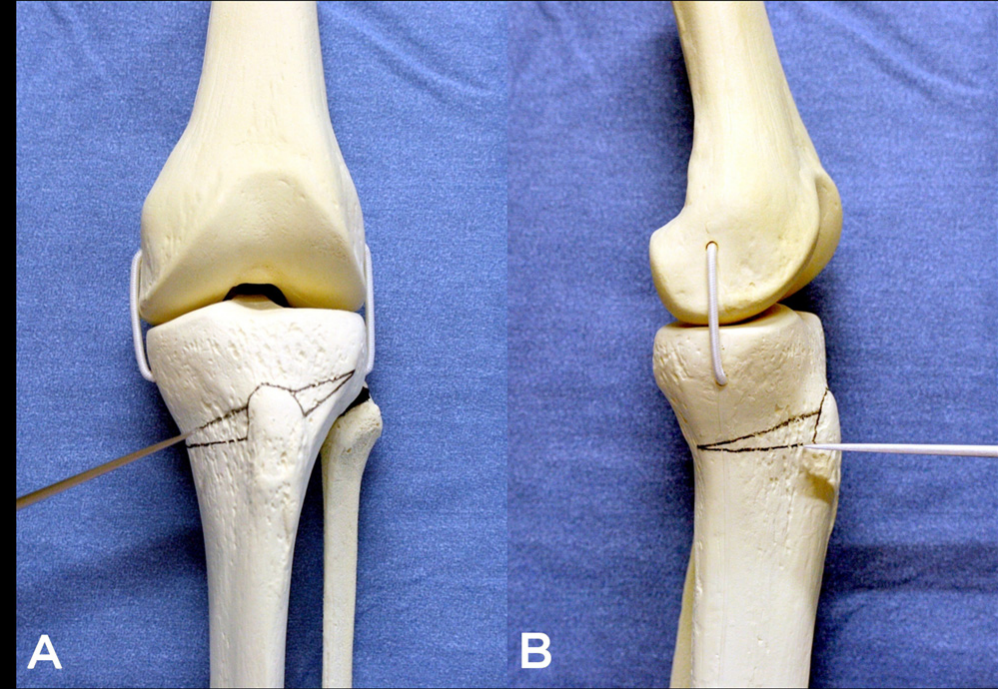
**Photographs showing the osteotomy procedures**. The black lines on the sawbone knee designed osteotomy line. First osteotomy was started from the medial cortex in the lateral direction (A). A second osteotomy was performed in the anterior-posterior direction to remove the wedge shaped tibial bone (B).

**Figure 4:**
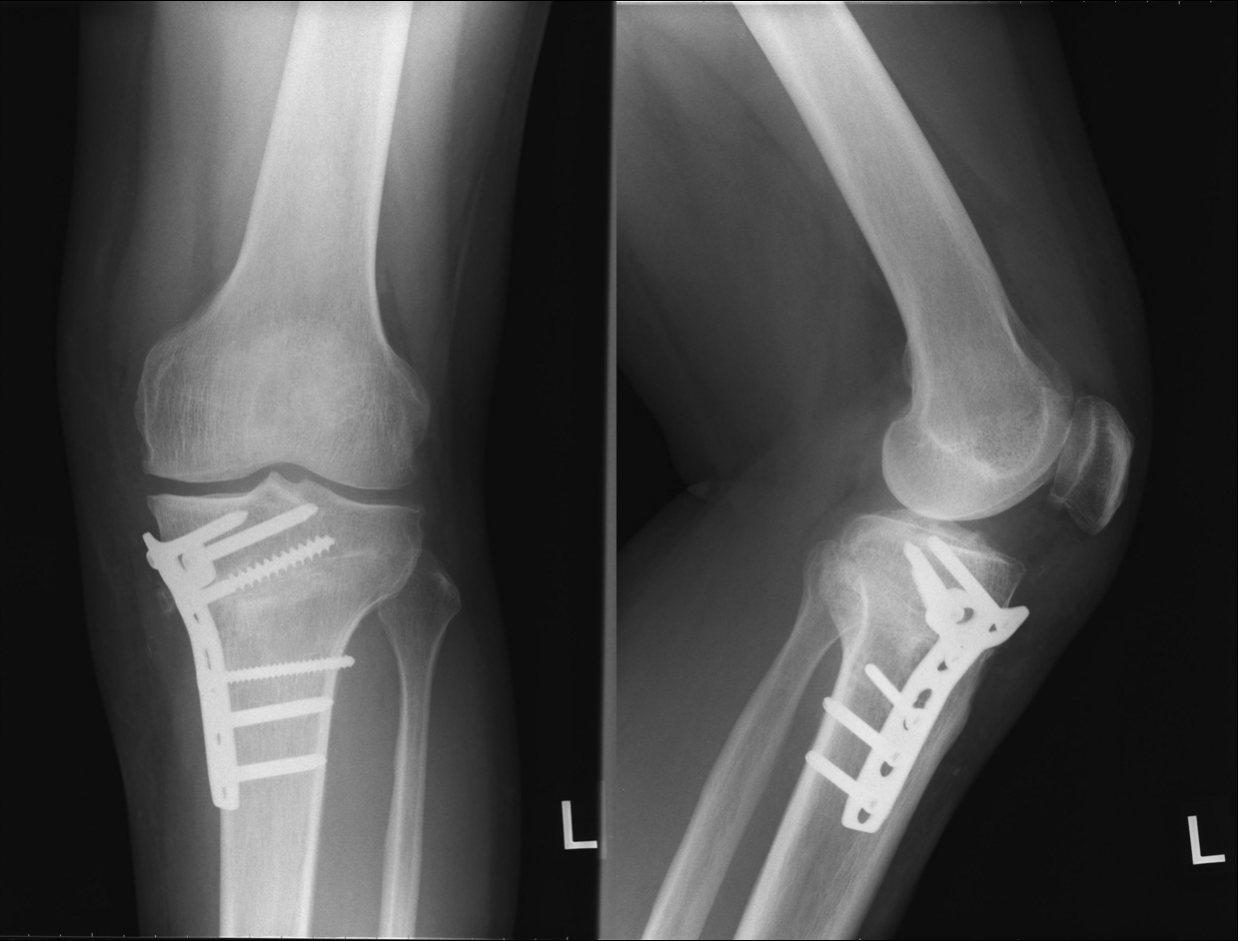
**Anteroposterior (left) and lateral radiographs (right) after the closing-wedge HTO**. The femorotibial angle was 173 degrees and posterior tibial slope was 8 degrees.

In January 2007, the patient first visited our institution for a second opinion, because he was recommended for another revision surgery to convert to total knee arthroplasty by the former hospital. He was unable to walk without a cane due to the left anteromedial knee pain. Physical examinations revealed quadriceps atrophy and no ligamentous laxity of the left knee. The range of motion was 140 degrees of flexion and -20 degrees of extension in the left knee, and 150 degrees of flexion and 0 degree of extension in the right knee. Severe left knee pain was observed with extension. The radiographs demonstrated complete bony union at the osteotomy site, a femorotibial angle of 169 degrees and posterior tibial slope of 20 degrees on the left side (Fig. 1), and 178 degrees and 8 degrees on the right side. The mechanical axis of the left leg passed through the middle of the lateral femorotibial compartment. We concluded that the increased posterior tibial slope limited his knee extension, and that was the cause of the pain. We discussed with the patient the treatment options to reduce the knee pain that was his chief complaint. Since the patient was young and had a physically demanding job, we choose a medial closing wedge osteotomy to recorrect the posterior tibial slope rather than conversion surgery to total knee arthroplasty. Under arthroscopic examination, the transplanted cartilage on the medial femoral condyle was preserved; however, the medial meniscus was down-sized by the prior debridement. Degeneration of cartilage was evident due to impingement of the anteromedial edge of tibial plateau and medial condyle of femur with the extension of knee. The cartilage fibrillation observed on the medial femorotibial compartment was classified as grade III according to a modified Outerbridge classification [13]. The patellofemoral joint showed no arthrosis change, and lateral femorotibial joint surface had normal appearance, confirming a suitable indication for realignment osteotomy. A longitudinal skin incision was made on the previous operative scar over the anteromedial aspect of the tibia. The pes anserinus covered with scar tissues was dissected, and the superficial medial collateral ligament was exposed and detached from the tibia. The osteotomy guide wires were then inserted from the medial tibial cortex and were stopped at 1 cm short of the lateral cortex and 1 cm below the joint line. Two additional parallel guide wires were inserted to the proximal and distal of osteotomy line directed from anterior to posterior in the sagittal plane (Figs. 2-A). These guide wires were used to estimate the correction angle of the posterior tibial slope. The osteotomy was performed, first from the medial cortex in the lateral direction according to the osteotomy guide wires, and second in the anteroposterior direction to remove the tibial bone in wedge shape and obtain full extension of the knee (Fig. 3-A, B). The angular correction of the posterior tibial slope was achieved by closing the bony space that was created by osteotomy, applying a knee extension torque manually. At the end of the corrective procedure, the 2 guide wires made an angle of 15 degrees and full extension of the knee was obtained (Fig. 2-B). Finally, a locking plate (LCP T-plate, Synthes^®^) was placed on the anteromedial tibia and fixed with 4 locking and 2 unlocking screws. Soft tissue releases were not performed due to the chronicity of the patient's flexion contracture. The postoperative radiographs showed 172 degrees of femorotibial angle and 8 degrees of posterior tibial slope (Fig. 4). No immobilization was applied and range-of-motion exercise using a continuous passive motion machine was started from the day after the surgery. Partial weight bearing was permitted at 4 weeks and full weight bearing was achieved at 6 weeks. The patient returned to pain-free daily life including running and stair ascending and descending with -5 degrees of extension and 135 degrees of flexion. The bone union was completed and the postoperative alignment was maintained for 2 years after the revision osteotomy. The knee score of the American Knee Society [14] was improved from 49 points of knee score and 35 points of functional score to 95 points and 90 points, respectively.

## Discussion

The HTO results in alignment changes in both the coronal and the sagittal plane. Several studies have shown that performing a HTO resulted in an undesirable change of the posterior tibial slope. Brouwer reported that mean posterior inclination of tibial plateau angle was increased 2.37 degrees in the opening-wedge HTO group, whereas it was reduced 3.77 degrees in the closing-wedge HTO group [8]. In opening-wedge HTO, anterior plate placement increased posterior tibial slope by an average of 6.6 degrees compared with posterior plate placement [15]. To maintain the normal tibial slope in the opening-wedge osteotomy, the opening gap at the tibial tubercle should be approximately one half of the opening gap at the posteromedial tibia because the proximal tibial shaft has a triangular cross-section [16]. Song recommended that the osteotomy should be fixed with 2 opening-wedge fixation plates, as the anterior plate was 2 to 4 mm shorter than the posterior plate [9]. For successful results in HTO, all changes in three-dimensional alignment should be monitored during surgery. Yamamoto showed availability of a computer navigation system in opening-wedge osteotomy and reported that the posterior tibial slope increased only 0.4 degrees after opening-wedge osteotomy [17]. Further development of the navigation systems for HTO may enable more accurate control for both coronal and sagittal alignment concurrently.

For recurrent knee pain after the HTO, most articles describing revision using a total knee arthroplasty, showed excellent or good results in more than 80% of the patients [18] and successful results comparable to the primary total knee arthroplasty [19]. A few reports described repeated osteotomy [20-22]. Hernigou reported that varus deformity can recur in the long term, but a second osteotomy can reproduce the same effects as the first [20]. Tsuda reported a case of revision osteotomy with the opening-wedge technique to treat early recurrence of varus alignment after a closing-wedge osteotomy [21]. These reports were focused on correction of the femorotibial angle in the coronal plane. To our knowledge, our case report is the first in the English-language literature to describe revision osteotomy using a closing wedge technique to correct the increased posterior tibial slope after failed opening-wedge HTO. We performed closing-wedge osteotomy from the medial approach to correct posterior tibial slope using a surgical technique with two guide wires for monitoring the sagittal angular correction. The posterior tibial slope was improved from 20 degrees to 8 degrees, close to 9 degree of that in the contralateral knee. However, the osteotomy removing the anteromedial cortex and the medial placement of the fixation plate resulted in the alignment change in the coronal plane, increasing the femorotibial angle from 169 degrees to 172 degrees. Since the mechanical axis still passed through the lateral tibial eminence in the whole leg radiograph, we believed that the effect to relieve the weight bearing load in the medial tibiofemoral joint had been maintained.

The presented patient may be a rare case who has more than 10 degrees of increase in the posterior tibial slope after the opening-wedge HTO. It is suggested, however, that surgeons should make effort to avoid excessive increase of the posterior tibial slope which might be one of the complications requiring revision surgery after the opening-wedge HTO. We realize that the usefulness of data from a single case is limited and that repeated osteotomy with this technique after opening-wedge technique may not be appropriate for all failed opening-wedge HTOs. For this patient, there was another treatment option with conversion to a total knee arthroplasty. However, we believed that this procedure would be the best way to decrease increased posterior tibial slope which could cause a loss of knee extension and pain. Also the patient was young and had a high activity level with heavy labor at work. Therefore, we chose the revision osteotomy to correct the excessive posterior tibial plateau inclination. The experience of this case suggested that repeated osteotomy would be one possible procedure for this type of failure in opening-wedge HTO.

## Content

Written informed consent was obtained from the patient for publication of this case report and accompanying images. A copy of the written consent is available for review by the Editor-in-Chief of this journal.

## Competing interests

The authors declare that they have no competing interests.

## Authors' contributions

YI and ET carried out the surgical treatment and AF and HT contributed to the follow-up examinations in an out-patient clinic. They also conceived of the study, and participated in its design and coordination. All authors read and approved the final manuscript.
